# Feasibility and efficacy of an acceptance and mindfulness-based group intervention for young people with early psychosis (Feel-Good group)

**DOI:** 10.3389/fpsyt.2022.943488

**Published:** 2022-09-16

**Authors:** Laura von Hardenberg, Karolina Leopold, Nikola Stenzel, Michèle Kallenbach, Navid Aliakbari, Andreas Bechdolf, Stephanie Mehl

**Affiliations:** ^1^Department of Psychiatry, Psychotherapy and Psychosomatic Medicine Incorporating Frühinterventions- und Therapiezentrum (FRITZ) and Soulspace, Vivantes Hospital am Urban and Vivantes Hospital im Friedrichshain, Charité – Universitätsmedizin Berlin, Berlin, Germany; ^2^Department of Psychiatry and Psychotherapy, Carl Gustav Carus University Hospital, Technische Universität Dresden, Dresden, Germany; ^3^Department of Psychology, Psychologische Hochschule Berlin (PHB), Berlin, Germany; ^4^Orygen, National Centre of Excellence in Youth Mental Health, University of Melbourne, Melbourne, VIC, Australia; ^5^Faculty of Medicine, Department of Psychiatry and Psychotherapy, Center for Mind, Brain and Behavior, Philipps-University Marburg, Marburg, Germany; ^6^Department of Health and Social Work, Frankfurt University of Applied Sciences, Frankfurt, Germany

**Keywords:** mindfulness-based therapy, early psychosis, group therapy, inpatient treatment, emotion regulation, early intervention

## Abstract

**Background:**

Over the last decade, researchers have sought for alternative interventions that have better treatment effects than Cognitive Behavioral Therapy (CBT) when treating psychotic symptoms. Mindfulness-based interventions have been a proposed alternative to CBT, yet research regarding its feasibility, acceptance and effectiveness is lacking when treating individuals with early psychosis in inpatient settings.

**Objective:**

Before conducting a large-scale randomized-controlled trial (RCT), this pilot study evaluated the feasibility and the potential efficacy of a mindfulness-based inpatient group intervention that targets emotion regulation in patients with early psychosis, and thus indirectly improving psychotic symptoms.

**Methods:**

A pre–post study was performed. Thirty-six patients with early psychosis treated at the specialized inpatient treatment “Frühinterventions- und Therapiezentrum; FRITZ” (early intervention and therapy center) received eight group therapy sessions. Assessments were performed at baseline, after 8 weeks post treatment and at follow-up after 16 weeks.

**Results:**

Rates of patients who participated in the study suggests that a mindfulness-based group therapy is highly accepted and feasible for patients with early psychosis being treated in an inpatient ward. Friedman analyses revealed significant changes in the primary outcomes of emotional goal attainment (Goal 1: *W* = 0.79; Goal 2: *W* = 0.71) and psychotic symptoms (PANSS-T: *W* = 0.74). Significant, albeit small, effect sizes were found in patients’ self-perception of emotion regulation skills (ERSQ: *W* = 0.23).

**Discussion:**

We found favorable findings regarding the feasibility and acceptance of the Feel-Good mindfulness-based intervention. Results of the study provide a basis for an estimation of an adequate sample size for a fully powered RCT that needs to be conducted to test whether Feel-Good is effective in the inpatient treatment of psychotic symptoms for individuals with early psychosis.

**Clinical trial registration:**

[https://clinicaltrials.gov/ct2/show/NCT04592042], identifier [NCT04592042].

## Introduction

Cognitive Behavioral Therapy, particularly Cognitive Behavioral Therapy for psychosis (CBTp), is an intervention commonly used when treating psychotic disorders and is highly recommended by numerous international guidelines (1, 2). CBTp identifies and focuses on changing negative appraisals and unhelpful coping strategies, in order to reduce distress, develop and/or improve coping strategies and improve everyday functioning (3). The intervention is usually delivered in a structured and stepwise manner that entails engagement, assessment, intervention, wellness, and relapse planning. Key components of CBTp are (1) a strong and robust therapeutic alliance, in which the normalizing attitude of the therapist toward symptoms is of importance, and (2) an individualized approach based on the needs of the patients (4). The cognitive aspect of CBTp aims to help people with psychosis identify and monitor their thoughts and assumptions throughout certain situations, thus localizing the sources of distress. The goal is then to evaluate and change these thoughts and assumptions by drawing on objective external evidence (5). The behavioral aspect of CBTp targets coping skills and reducing problematic behaviors (i.e., social challenges, using drugs or alcohol or other avoidance techniques) that are specifically linked to psychosis. CBTp. Whereas CBTp has been found to be more efficacious than routine care or other psychological interventions, the effect on the reduction of positive symptoms, overall and negative symptoms, response to treatment, quality of life, and functioning remains small (6–10).

One possible way to achieve stronger overall intervention effects that have long-lasting effects on psychotic symptoms may be to include more “third-wave” therapies in the treatment of psychotic patients. Third-wave therapies (or mindfulness-based therapies) entail a diversity of interventions that focus on different aspects of mindfulness and metacognitive knowledge on thoughts, emotions and behavior. Some interventions focus on awareness and attention (i.e., meditation-based practices), some focus on acceptance and detachment (i.e., acceptance and commitment therapy; ACT), whereas others focus on kindness and compassion (i.e., compassion-focused therapy) and on improving metacognitive knowledge on one’s own thought processes (worrying) (i.e., metacognitive therapy). Though different techniques are used, all third-wave interventions target similar basic principles, which are non-judgmental awareness, self-compassion, acceptance, and defusion (the ability to observe and experience one’s thoughts and feelings without automatically identifying with them) (11). The goal of these interventions is to focus on an individual’s relationship with, and responses to, experiences and symptoms rather than to change them. Thus, by enhancing the psychological flexibility of patients with psychosis, it prevents avoidance strategies toward psychotic symptoms and may help handle negative emotions (i.e., distress) in patients who experience acute psychotic phases.

Numerous meta-analysis and systematic reviews have examined the feasibility and effectiveness of third-wave interventions for psychotic symptoms. Of note is the high heterogeneity regarding outcomes, training protocols and objectives used, thus, there is a large disparity between findings. Two meta-analyses of third-wave interventions for psychosis have found small-to-moderate treatment effects on positive symptoms, thus, resembling the effect sizes of CBTp (12, 13). On the other hand, one systematic review found no positive effect on the reduction of distress relating to auditory hallucinations when utilizing third-wave interventions (14). Similarly, whereas one meta-analysis of eight randomized-controlled trials (RCTs) found that third-wave interventions had no effect on negative symptoms, a different meta-analysis showed higher effects on negative symptoms than positive symptoms (13, 15). Thus, more research is needed to assess the feasibility and effectiveness of third-wave interventions for psychotic symptoms.

More research is especially needed when examining the effects that third-wave interventions have on individuals with early psychosis (EP; the first 5 years after onset of the first psychotic episode) (16). As diagnoses frequently change over the course of an illness trajectory, EP encompasses a wide range of psychotic disorders, including schizophrenia, substance-induced psychotic disorder, schizoaffective, as well as affective psychotic disorders, and thus may have different treatment needs compared to patients with longer history of illness (17–23). Furthermore, EP has been deemed a critical period in which biological and psychological changes are most extensive, as well as a critical time to build a stable social identity and form relationships (24). Therefore, it is essential to adapt psychological interventions to the specific needs of patients with EP in this critical period.

Research has found that besides psychotic symptoms, patients with EP frequently suffer from other conditions such as low self-esteem, rumination, negative emotions (e.g., anxiety and depression) (25–33). Prior research suggests that low self-esteem, rumination, and negative emotions (e.g., anxiety and depression) are important mediators involved in the development and maintenance of psychosis, as well as in the distress associated with it (34–36). Furthermore, it was found that emotion regulation (ER) was markedly impaired for people with psychotic disorders, as they reported a greater use of more putatively maladaptive strategies (i.e., rumination) and less frequent use of efficacious strategies (i.e., cognitive flexibility) (37). Also, individuals with psychosis are less willing to experience negative emotions when pursuing meaningful activities compared to controls, as well as having more difficulties identifying, accepting, and understating their emotions (38). These findings highlight the difficulties individuals with psychosis, including EP, have in terms of ER; difficulties that have been associated with numerous negative consequences, such as more severe psychotic experiences, poorer social functioning, reduced emotional well-being, and increased psychological distress (39–47). Personal accounts of individuals with psychosis express the desire to obtain more support in dealing with negative emotions in their therapies (48–52). Thus, specifically targeting the mediator ER in therapeutic interventions for individuals with EP may be a plausible way to achieve a stronger overall intervention effect on psychotic symptoms.

To our knowledge, only one meta-analysis including eight RCTs exists that examines the feasibility and effectiveness of third-wave interventions on individuals with EP (53). All studies included were small-scale pilot or feasibility studies who reported favorable findings regarding the feasibility and acceptance of mindfulness-based interventions. However, as this field of research has been less extensively explored up until now, the eight RCTs differed in terms of utilizing various models and formats/lengths and did not allow for preliminary conclusions to be drawn. In fact, the meta-analysis concluded that there was insufficient evidence available and so recommendations regarding the incorporation of mindfulness-based interventions into routine care cannot be made. Of note, is that none of the eight studies solely focused on group therapy for individuals with EP in inpatient settings. Thus, more research is needed to gather more conclusive evidence regarding the feasibility and effectiveness of group third-wave interventions in inpatient settings for individuals with EP. Implementing third-wave interventions in the treatment of individuals with EP as early as possible in their treatment plan is vital, as a lot of patients never reach outpatient settings after being discharged from inpatient settings due to different reasons (i.e., long waiting periods for outpatient therapy) (54, 55).

To summarize, there is a lack of research examining the feasibility of a mindfulness-based group intervention in an inpatient setting for patients with EP. Large-scale interventional studies need to be conducted with individuals with EP, especially in inpatient settings, to gather more conclusive evidence on the effects of group mindfulness-based interventions. Therefore, our primary aim was to assess whether a mindfulness-based inpatient group intervention targeting ER specifically is feasible for and accepted by patients with EP in an inpatient setting. A secondary aim was to gather initial evidence for the potential efficacy of a mindfulness-based inpatient group. We did this by exploring whether targeting and improving strategies of ER indirectly led to: (1) improvements in subjective emotional goal attainment and reductions of psychotic symptoms and general psychopathology (primary outcome variables), (2) reductions of other clinical symptoms (i.e., depression) and improvements in everyday functioning (secondary outcome variables), as well as (3) improvements in ER skills (putative mediator). Findings from this study should help inform and shape a large-scale RCT intervention study for individuals with EP in inpatient settings.

## Materials and methods

### Study design

The study utilized a pre–post design to investigate the feasibility and efficacy of an 8-week mindfulness-based group intervention in a specialized inpatient and day-care unit for EP. Assessments were performed at the start of therapy (pre-therapy), 8-weeks-post therapy, and at 16-week follow-up (FU) period.

### Ethical approval

The study was reviewed and approved by the Ethics Committee of the Psychologische Hochschule Berlin. Participants and/or the participants’ legal guardian/next of kin provided written informed consent to participate in this study. The study was registered at ClinicalTrials.gov (Identifier: NCT 02787122).

### Participants

#### Inclusion and exclusion criteria

Participants were recruited in the specialized inpatient and day-care treatment ward “Frühinterventions- und Therapiezentrum; FRITZ” (early intervention and therapy center) in Berlin. Inclusion criteria were the following: (i) aged between 17 and 65 years, (ii) diagnosis of a schizophrenia, schizoaffective disorder, psychotic disorder, bipolar disorder with psychotic symptoms using the ICD-10, (iii) onset of the first psychotic episode or first presentation to mental health services in the last 5 years, (iv) an estimated verbal intelligence score of ≥80 in the German Mehrfachwahl-Wortschatz-Intelligenztest [MWT-B; (56)], (v) absence of current suicidal tendencies reported in the Structured Clinical Interview for DSM-5 [SCID-5; (57)], (vi) no diagnosis of dementia within the last 6 months as reported in the SCID interview, and (vii) proficient use and comprehension of the German language.

#### Recruitment

Participants were recruited between November 2020 and November 2021 directly in the Vivantes hospital’s specialized ward for patients with EP (“FRITZ”). The group intervention commenced on the FRITZ ward in January 2021; FU assessments were completed in January 2022. Potentially eligible patients and legal guardians for patients under 18 received an in-depth information session with a clinical psychologist (LH) who informed them about the study and its duration and focus. If patients and, when necessary, their legal guardians were interested in participation, written consent was obtained, and two 2-h diagnostic assessment appointments were scheduled to assess eligibility. All primary and secondary outcome measures, as well as the putative mediators, were assessed at pre-, 8-weeks- post-, and 16-weeks-FU- assessment.

### Intervention

#### Mindfulness-based intervention for patients with early psychosis

The aim the mindfulness-based intervention is to target the putative causal factors of psychotic symptoms such as ER and emotional well-being, rather than the psychotic symptoms directly per se. On FRITZ, the mindfulness-based intervention (named “Feel-Good”) consisted of 8 therapy sessions (50 min) offered weekly or twice-weekly for 6–8 patients over 8 weeks. The Feel-Good group was offered in addition to the usual treatment provided on FRITZ (pharmacology, individual and group therapy, and socio-therapeutic approaches; [see Siebert (58)]. Feel-Good was an open-enrolling group, so patients were able to join the group therapy sessions at any time and then participated at eight consequent sessions. Feel-Good entails numerous third-wave approaches, including Acceptance and Commitment Therapy (59), Emotion-Focused Therapy (60), Compassion-Focused Therapy (61), and Schema Therapy, in addition to some classic CBT interventions, such as psychoeducation and self-monitoring techniques. Supplementary Table 1 in the supplement summarizes a brief overview of the modules utilized in the Feel-Good intervention. For a more in-depth and detailed description of the interventions see Mehl et al. (62).

#### Therapists and raters

Therapists were two clinical psychologists (M.Sc.) who were enrolled in their final year of German postgraduate training to become certified CBT therapists and have worked on the FRITZ ward for at least 3 years. Both therapists underwent an additional training (8 h) on the Feel-Good intervention and received supervision by one of the study PIs (SM) once a month. Study assessments and ratings were conducted by an independent psychologist (M.Sc.) who received training and had experience with the utilized interviews and questionnaires from prior research projects.

### Measures

#### Screening

At baseline, the MWT-B was used to determine an estimate of verbal intelligence, whereas the SCID was used to determine diagnoses (that were then transferred to ICD-10). An adapted version of the Nottingham Onset Schedule (63) was used to assess the duration of untreated psychosis (DUP).

#### Feasibility and acceptance of therapy

Regarding treatment feasibility/acceptance, we assessed attrition rates and reasons for dropouts.

#### Primary outcome measures

The *Goal Attainment Scale* [GAS; (64)] is an interview used to help patients formulate at least two individual therapy goals at the first assessment. As the Feel-Group intervention was emotion-oriented, the goals for the GAS were also emotion-oriented. Patients were asked to identify emotions they struggled with, felt overwhelmed by, or had difficulties showing. In the interview, the individual emotions named by the patients were explored by the interviewer with the patients to identify and help formulate patients’ specific emotional therapy goals (i.e., “I want to be less angry” would be explored and a more specific goal would be set, such as “I would like to find better strategies on how to deal with my anger. At the moment I tend to release it by screaming in public or hitting things, such as the wall.”). After the individual emotion therapy goals were set, patients were asked to rate on a 5-point Likert scale whether their individual goals are met in the present moment. The scores ranged from “much less than expected” (−2) to “more than expected” (+2). Later, the GAS was used to assess to what extent patients estimated whether their individual goals were achieved at 8-weeks post-treatment and 16-week-FU.

The *Positive and Negative Syndrome Scale* [PANSS; (65)] is a semi-structured interview used to assess psychotic symptom severity. The interview is divided into three scales and is scored using a 7-point Likert scale [PANSS positive scale (7 symptoms: range 7–49), PANSS negative scale (7 symptoms, range: 7–49), and PANSS general psychopathology scale (16 symptoms, range: 16–112)]. For this study, the total PANSS score was used as primary outcome. In addition, the subscales were used as secondary outcome variables.

#### Secondary outcome measures (symptoms and functioning)

The *Calgary Depression Scale for Schizophrenia* [CDSS; (66)] is a semi-structured interview utilized to assess depressive symptoms in people with schizophrenia. A total CDSS score was computed and used by adding all items together (range 0–21).

The *Role Functioning Scale* [RFS; (67)]; German version; (68) measures the level of functioning in four different domains: working productivity, independent living and self-care, immediate social network relationships (family and friends), and extended social network relationships (other social contacts). There are five items, which were used to compute a mean score (range 12–48). Higher scores indicate higher social functioning.

The *Paranoia Checklist* [PCL; (69)]; German version; (70) is an 18-item self-report questionnaire that examines the frequency, distress, and conviction of paranoid delusions/suspicious thoughts. Items are answered and rated using a 5-point Likert scale. Higher scores indicate more paranoid/suspicious thoughts present. All three subscale scores were utilized in this study.

The *Psychotic Symptom Rating Scale* [PSYRATS; (71)] is made up of two different scales: delusions and hallucinations (PSYRATS-D and PSYRATS-H, respectively). Both scales are based on a semi-structured interview that assess different aspects of the psychotic symptoms, including the amount and duration of preoccupation, conviction, disruption of daily life, and amount and intensity of distress. PSYRATS-H is comprised of 11 items (range: 0–44), whereas PSYRATS-D is comprised of 6 items (range: 0–24). All items are answered on a 5-point Likert scale.

The *Peters et al. Delusions Inventory – short version* [PDI-21; (72)]; German Version; (73) is a 21-item questionnaire split into four subscales: endorsed delusions, delusional beliefs, frequency of delusional beliefs and conviction of delusional beliefs. Participants are first asked whether they endorse a delusional belief and are then asked to rate the frequency, preoccupation, distress and conviction of that belief on a 7-point-Likert scale. A PDI Total score was computed by summing up the subscales (range 0–336).

Three different measures (PCL, PSYRATS, and PDI-21) to assess delusions were included in this study to examine different aspects of delusions. The PCL assesses paranoia specifically, which is one of the main symptoms of delusions. The PDI focuses on whether delusional beliefs are present, how frequently it occurs and how convinced the participant is of that belief. The PSYRATS covers some of the same aspects that the PDI-21 does (preoccupation and conviction of delusional beliefs), yet, it also examines the intensity of distress and decline in functioning attributed to delusions.

#### Putative mediators (emotion regulation)

The *Beliefs about Stress Scale* [BASS; (74)] is a questionnaire exploring three dimensions of stress: negative stress beliefs, positive stress beliefs, and controllability of stress [BASS-N: range (8–32); BASS-P: range (3–12), and BASS-C: range (4–16), respectively].

The *Emotion Regulation Inventory* [ERI; (75)] assesses a patients’ ability to utilize strategies to regulate negative (ERI-NE) and positive (ERI-PE) emotions. There are 47 items that are answered and scored using a 5-point Likert scale (0 = “never applies” to 4 = “always applies”).

The *Emotion Regulation Questionnaire* [ERQ; (76)] is a 10-item questionnaire designed to measure the tendency individuals have to regulate their own emotions in terms of Cognitive Reappraisal (ERQ-C) and Expressive Suppression (ERQ-S). All items are answered on a 7-point Likert-scale ranging from 1 (strongly disagree) to 7 (strongly agree) (range: 10–70).

The *Rosenberg Self-Esteem Scale* [RSE; (77)] is a 10-item scale that assesses global self-worth. It measures both positive and negative feelings about the self. All items are answered using a 4-point Likert scale that ranges from 1 (strongly disagree) to 4 (strongly agree) (range: 0–30).

*Self-Compassion Scale* [SCS; (78)]; German Version; (79) is a 26-item questionnaire that measures six areas of self-compassion: self-kindness, self-judgment, common humanity, isolation, mindfulness, and over-identification. The total SCS score is computed by summing up all the mean values of the subscales and calculating the total mean, with higher scores indicating high self-compassion (range: 1–5).

The *Emotion Regulation Skills Questionnaire* [ERSQ; (80)] is a self-report on ER skills with 27 items rated on a 5-point Likert scale (range 0–108). In the present study, the total score is used.

### Changes to the study

The study was registered at ClinicalTrials.gov (Identifier: NCT: NCT04592042). The following changes were made after the start of the study. We renamed the intervention from emotion focused CBT (CBT-E) to Acceptance and Mindfulness-Based Group Intervention, because this reflects the applied strategies more precisely and allows for comparisons with international studies, which apply these strategies. For the statistical analysis plan, a power analysis was conducted based on the assumption that *t*-tests will be used to measure changes from pre- to post-group intervention in the primary outcome variables. However, after data retrieval was concluded we wanted to compare all three-assessment time points for the primary outcome variables in one analysis, which is why we decided to utilize the Friedman test. We decided against conducting another power analysis, as they are not recommended for pilot projects due to large confidence intervals (CIs) around effects in smaller samples (81). In addition, we exchanged the *Illness Perception Questionnaire for Schizophrenia* [IPQS; (82)] and instead used the BASS as the authors of the IPQS scale never answered our request to utilize the questionnaire in German. Also, the self-report questions on acceptance of the intervention (68) were included in a semi-structured interview that was performed by an independent student. The transcripts and results will be published in an additional manuscript on subjective efficacy of the intervention.

### Dropouts

**FIGURE 1:**
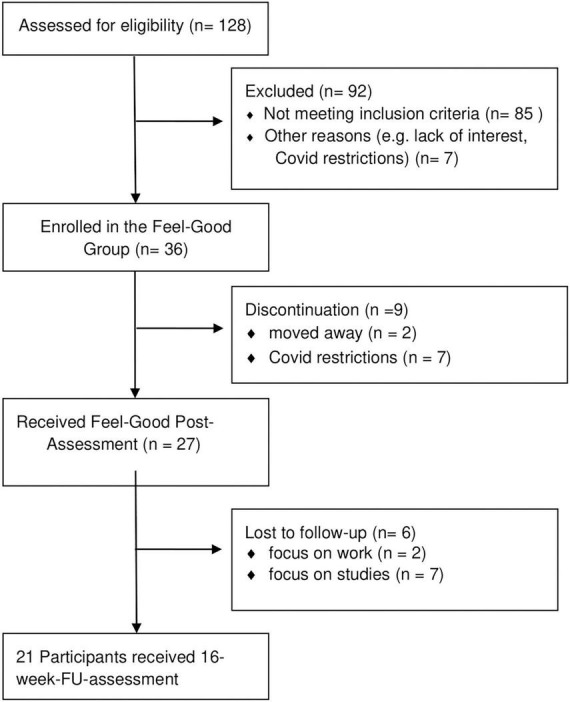
Flowchart of patient recruitment.

Based on the study protocol, we aimed to recruit 30 patients in total. Due to German COVID restrictions and two patients moving away (see Figure 1), nine patients were discharged from the hospital before baseline data was retrieved. Therefore, additional 9 patients were recruited (*N* = 36), thus, pre- and post-data was obtained from 27 patients in total and used in our analyses. It was determined that all participants had to partake in at least six of the eight therapy sessions to remain in the study as completers. Six out of eight sessions were deemed essential as there were numerous factors influencing attendance rates that were not attributed to the motivation or interest of the participants: illness (i.e., Flu or COVID), side-effects of medication (i.e., drowsiness and sleepiness), other group therapies or individual sessions being completed parallel to the Feel-Good group, or outside appointments with government or housing authorities. Six patients (22%) dropped out between 8-weeks-post-treatment and 16-weeks-FU assessment (did not attend assessment session at 16-weeks-FU), as they were focused on reintegrating in their studies or job.

### Analyses

Regarding treatment feasibility and acceptability, attendance rates and reasons for drop-out were assessed.

We also assessed changes in scores across the primary and secondary outcomes, as well as among the putative mediators, over all three-assessment time points. Due to 22% of missing data at FU, multiple imputation was used for FU data solely (83). As missing data at FU was below 60%, we imputed a total of 40 datasets as recommended by Graham et al. (84). Imputed variables included all primary outcome variables with the exception of the GAS scores, as well as all secondary and putative mediators. Scores at pre- and 8-weeks-post-treatment interventions for each variable were used as predictors to impute scores at FU.

As the data was not normally distributed, differences in primary outcomes, secondary outcomes and putative mediators at pre-, 8-weeks-post-treatment and 16-weeks-FU time points were calculated using a Friedman test (at three assessment points: pre-treatment, 8-weeks-post-treatment, and 16-weeks-FU). In case of significant main effects, post-hoc analyses were performed using the Wilcoxon test. As three follow-up comparisons were included in the paired Wilcoxon test, we adjusted the *p*-level to 0.017 (Bonferroni correction) (85). The effect size Kendall’s W was computed and can be interpreted accordingly: as a small (W > 0.2), moderate (W > 0.5), and large effect (W ≥ 0.8) (86).

Also, sensitivity analyses were conducted to assess whether any discrepancies occurred in the findings between imputed and non-imputed data. To do this, all analyses conducted for imputed data were also conducted for the non-imputed data.

Lastly, we conducted an exploratory analysis to examine whether medication has an indirect influence on the significant changes found in our primary outcome variables or whether these changes can be attributed mainly to the Feel-Good intervention. To do this, we conducted a non-parametric mediation analysis using the “Causal Mediation Analysis” package for R (87). Post scores of the primary outcomes (8-weeks-post-treatment) were used as dependent variables and pre-scores of the outcome variables were used as predictor variables. Point estimates and 95% bias-corrected CI were generated using the default 1,000 simulations.

All quantitative analyses were conducted using R (88).

## Results

### Sample characteristics

See Table 1 for socio-demographic and clinical data.

**TABLE 1:** Sociodemographic and clinical characteristics of participants at baseline.

Sociodemographic baseline characteristics	Sample (*n* = 27)		Clinical baseline characteristics	Sample (*n* = 27)	
			
	*M*/*N*	SD (%)		*M*/*N*	SD (%) (range)
Age (years)	23.33	4.83	Primary diagnosis^b^		
Gender (female)	11	40.7	Schizophrenia	11	40.7
Marital status			Schizoaffective disorder	1	3.7
Single	26	96.3	Manic episode with psychosis	1	3.7
Relationship	1	3.7	Bipolar disorder	3	11.1
Education (years)	13.06	2.13	Drug-induced psychotic disorder^c^	11	40.7
Nationality			Amphetamine	1	3.7
German	23	85.2	Cannabis	6	22.2
Turkish	1	3.7	Hallucinogens	1	3.7
Other	3	11.1	Multiple drug use	3	11.1
Number of psychotic episodes			Comorbid diagnosis	18	66.7
1 episode	9	33.3	Past psychotropic medication	16	59.2
2 episodes	9	33.3	Current psychotropic medications	6	12
3+ episodes	9	33.3	AP	27	100
Family history of mental illness^a^	23	85.2	AD	1	3.7
Schizophrenia	6	22.2	MS	2	7.4
Bipolar disorder	3	11.1	CPZi^d^	485.56	382.73
Depression	19	70.4	DUP (days)	202.31	324.57 (4–1,460)
Alcohol dependency	9	33.3			
Drug dependency	4	14.8			
Anxiety	4	14.8			

M, mean; N, number; SD, standard deviation; AP, antipsychotics; AD, antidepressants; MS, mood stabilizers; CPZi, chlorpromazine index; DUP, duration of untreated psychosis.

*^a^*Family history of psychiatric illness includes both first and second generation family members.

*^b^*ICD-10 codes reported.

*^c^*Drug-induced psychotic disorder diagnosed at this time point, as psychotic symptoms occurred solely while consuming drugs. Diagnosis may change throughout the course of the illness.

*^d^*Chlorpromazine equivalent index based on Möller (98) and updated for newer Antipsychotics based on Schmauß et al. (99).

### Feasibility and acceptability

All of the 27 patients attended all sessions of the Feel-Good group intervention, with the exception of two patients (attended six of the eight sessions). There were no drop-outs between pre- and post-group intervention. Six patients (22%) dropped out between post-group and FU because they wanted to focus on their studies (*n* = 4) or their work (*n* = 2) and did not have time to attend the assessment time-point at 16-week-FU that took place during work hours/lectures.

### Changes in primary outcomes

#### Goal attainment scale

**FIGURE 2:**
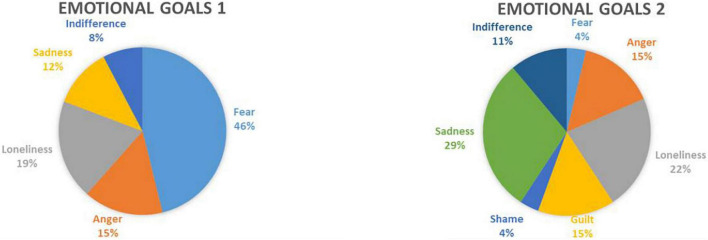
Emotions participants wanted to work on in the Feel-Good intervention.

From the individual emotional therapy goals of each patient, categories of main emotions reported were established solely for descriptive purposes (see Figure 2). Overall, most patients presented a change in the emotion fear as their most important goal for the intervention (44.4%), 29.6% chose the emotion sadness as their second most important emotion goal. As depicted in Table 2, there was statistically significant change over time for patients’ first and second individual therapy goals (GAS scale 1: *X*^2^ = 44.1, *p* < 0.01; GAS scale 2: *X*^2^ = 30.0, *p* < 0.01). Results of the post-hoc Friedman test revealed a significant change at post- and FU-assessment for the first individual goals (GAS scale 1: all *p* < 0.01). Regarding the second emotion intervention goal (GAS scale 2), there was significant change between pre- and post-treatment-assessment and between pre-treatment-assessment and FU-assessment (*p* < 0.01), but not between post-treatment-assessment and FU-assessment.

**TABLE 2:** Change in primary and main secondary outcome variables and putative mediators between pre-, post- and follow-up assessments (multiple imputation sample; *n* = 27).

Measure	T1 scores *M* (SD)	T2 scores *M* (SD)	T3 scores *M* (SD)	Timepoint differences			Effect sizes		Pairwise comparisons*					
						
									T1–T2		T2–T3		T1–T3	
											
				χ^2^	*df*	*p*	W	95% CI	Z	*p*	Z	*p*	Z	*p*
**Primary outcome variables**														
GAS 1	−1.44 (0.75)	0.11 (1.28)	0.79 (1.06)	44.1	2	<0.001	0.82	0.72-0.91	0	<0.001	–2.55	0.011	–3.97	<.002
GAS 2	−1.29 (0.78)	0.24 (1.3)	0.81 (1.17)	30.00	2	<0.001	0.71	0.60−0.87	–1.07	0.002	–0.36	0.740	–1.43	0.000
PANSS T	68.43 (18.12)	59.00 (16.19)	50.76 (14.73)	31.14	2	<0.001	0.74	0.59−0.86	–3.26	0.001	–3.39	<0.001	–4.017	<0.001
**Secondary outcome variables**														
PCL-F (P)	25.57 (17.00)	14.38 (15.68)	11.10 (14.70)	17.84	2	<0.001	0.41	0.17−0.70	–3.19	0.001	–2.10	0.036	–3.47	<0.001
PCL-C (P)	27.86 (18.52)	16.00 (15.56)	11.86 (14.06)	23.73	2	<0.001	0.46	0.25−0.70	–3.37	<0.001	–2.00	0.046	–3.62	<0.001
PDI T	67.38 (52.42)	34.00 (43.55)	21.71 (37.43)	38.4	2	<0.001	0.71	0.50−0.91	–3.87	<0.001	–3.09	0.002	–4.02	<0.001
PANSS P	18.15 (5.69)	13.15 (4.57)	10.56 (3.74)	34.9	2	<0.001	0.65	0.49−0.83	–3.78	<0.001	–4.27	<0.001	–3.75	<0.001
PANSS N	17.00 (8.30)	15.30 (5.54)	13.78 (5.16)	6.61	2	0.003	0.12	0.04−0.39	–1.22	0.223	–3.09	0.002	–1.81	0.071
PANSS G	35.04 (6.14)	31.04 (6.89)	26.68 (6.20)	30.6	2	<0.001	0.57	0.36−0.76	–2.54	<0.001	–4.40	<0.001	–3.47	<0.001
PSYRATS-D	14.62 (6.77)	7.62 (7.26)	5.86 (6.38)	35.1	2	<0.001	0.65	0.39−0.89	–4.04	<0.001	–2.52	0.012	–3.69	<0.001
PSYRATS-H	11.74(15.10)	6.48 (11.99)	4.99 (10.11)	16.0	2	<0.001	0.30	0.15−0.49	–2.39	0.017	–2.02	0.043	–2.67	0.008
RFS	38.52 (9.72)	39.19 (8.30)	42.91 (8.07)	9.77	2	<0.011	0.18	0.05−0.40	–0.45	0.681	–2.78	0.005	–2.21	0.027
CDSS	4.62 (3.28)	5.24 (3.66)	4.10 (4.21)	1.92	2	0.382								
PCL-D (P)	33.05(20.74)	19.81 (19.88)	17.71 (19.64)	4.53	2	0.104								
**Putative mediators**														
ERSQ (P)	57.30 (24.33)	59.48 (17.01)	63.10 (19.51)	10.7	2	<0.011	0.20	0.04−0.48	–0.534	0.594	–1.86	0.061	–1.63	–0.102
BASS-N (P)	23.43(6.08)	22.19 (4.78)	22.05 (4.20)	4.84	2	0.089								
BASS-P (P)	7.90 (3.83)	8.14 (2.18)	8.81 (2.93)	6.09	2	0.048								
BASS-C (P)	6.95 (2.44)	7.57 (1.66)	8.43 (1.81)	1.42	2	0.491								
ERI- NE (P)	36.33 (10.88)	41.00 (11.27)	40.05 (10.88)	4.84	2	0.089								
ERI-PO (P)	22.52 (10.07)	21.24 (9.85)	19.14 (7.20)	4.23	2	0.121								
ERQ-R (P)	22.33 (6.57)	24.76 (6.55)	25.90 (4.42)	3.88	2	0.144								
ERQ-S (P)	15.24 (6.62)	13.76 (5.29)	15.43 (4.42)	3.49	2	0.175								
RSE (P)	25.29 (3.07)	26.05 (1.66)	25.67 (2.37)	1.50	2	0.472								
SCS (P)	2.74 (.72)	2.91 (.47)	3.10 (.61)	4.67	2	0.097								

M, mean; SD, standard deviation; df, degrees of freedom; W, Kendall’s W; CI, confidence Interval; GAS, Goal Attainment Scale; PANSS, Positive and Negative Syndrome Scale; PANSS T, PANSS total score; PCL-F, Paranoia checklist frequency; PCL-C, Paranoia checklist conviction; P, self-report questionnaire; PDI, Peters et al. Delusions Inventory 21 total score; PANSS P, PANSS positive scale; PANSS N, PANSS negative scale; PANSS G, PANSS general psychopathology scale; PSYRATS-D, psychotic rating symptom scale delusions; PSYRATS-H, PSYRATS hallucinations; RFS, role functioning scale: RFS mean score, mean score of scales social network I, social network II, work and living; CDSS, Calgary Depression Rating Scale; PCL-D, Paranoia checklist distress; ERSQ, Emotion Regulation Skills Questionnaire; BASS-N, Beliefs about Stress Scale-Negative; BASS-P, Beliefs about Stress Scale-Positive; BASS-C, Beliefs about Stress Scale-Controllability; ERI-NE, Emotion Regulation Inventory–Negative; ERI-PO, Emotion Regulation Inventory-Positive; ERQ-R, Emotion Regulation Questionnaire-Cognitive Reappraisal; ERQ-S, Emotion Regulation Questionnaire-Expressive Suppression; RSE, Rosenberg Self-Esteem Scale total score; SCS, Self-Compassion Scale total score.

*Pairwise comparisons were only conducted for variables where the Friedman analysis showed significant effects.

#### Positive and negative syndrome scale total score

Results of the Friedman test revealed a statistically significant change over time regarding general psychotic symptoms (PANSS total score) (*X*^2^ = 31.14,*p* < 0.01). *Post hoc* pairwise comparisons revealed that significant changes were present between all assessment time points (*p* < 0.01; see Table 2).

#### Adverse events

There were no adverse events throughout this study.

### Secondary outcomes

#### Clinical symptoms

##### Psychotic symptoms

Results of the Friedman test revealed a statistically significant change over time for positive psychotic symptoms (PANSS-P: *X*^2^ = 34.9,*p* < 0.01) in general. Post-hoc pairwise comparisons revealed that significant changes were present at all assessment time points (*p* < 0.01). Results on the Friedman test for delusions specifically revealed a statistically significant change over time in most scales (PCL-F: *X*2= 17.84,*p* < 0.01; PCL-C: *X*^2^ = 23.73,*p* < 0.01; PDI: *X*^2^ = 38.4,*p* < 0.01; PSYRATS-D: *X*^2^ = 35.1,*p* < 0.01). *Post hoc* pairwise comparisons revealed that significant changes were present at nearly all post-assessment time points (*p* < 0.01; see Table 2), except for the Paranoia checklist frequency subscale (PCL-F) and the Paranoia checklist conviction subscale (PCL-C), where no statistically significant change between post-assessment and FU-assessment time points was revealed.

Hallucinations (PSYRATS-H) specifically also showed significant changes that remained over time(*X*^2^ = 16.0,*p* < 0.01). *Post hoc* pairwise comparisons revealed that significant changes were present between pre- and post-assessment, as well as between pre- and FU-assessment time points (*p* < 0.01; see Table 2). No statistically significant change between post-assessment and FU-assessment time points was found.

Results of the Friedman test regarding negative symptoms (PANSS-N) also revealed a statistically significant change over time (*X*^2^ = 8.71,*p* < 0.01). *Post hoc* pairwise comparisons revealed that significant changes were present between post- and FU-assessment (*p* < 0.01). No significant changes were found between pre- and post-assessment, nor between pre- and FU-assessment.

##### Depression and functioning

The results of the Friedman test for general psychopathology (PANSS-G) revealed a significant change over time (*X*^2^ = 29.5,*p* < 0.01). *Post hoc* pairwise comparisons revealed that significant changes were present at all assessment time points (*p* < 0.01). Results of the Friedman test revealed no significant change over time regarding depressive symptoms (CDSS). A statistically significant change over time was found for everyday functioning (RFS: *X*^2^ = 9.77,*p* < 0.01). *Post hoc* pairwise comparisons revealed that significant changes were present between post- and FU-assessment time points (*p* < 0.01; see Table 2). No significant changes were present between pre- and post-assessment, nor between pre- and FU-assessment.

#### Putative mediators (emotion regulation)

As depicted in Table 2, the ERSQ scale was the only scale that showed significant changes over time (*X*^2^ = 10.7,*p* < 0.01). Pairwise comparisons revealed no significant changes between any of the assessment time points. All other scale assessing different elements of ER (BASS, ERI, ERQ, RSE, and SCS) showed no significant changes.

### Sensitivity analyses

Differences in significant findings were found for non-imputed data regarding the negative symptoms of psychosis (PANSS-N:*X*2=5.84,*p* > 0.05) (see Supplementary Table 2). Furthermore, the non-imputed data revealed an increase in the significance of ER (ERQS:*X*2=9.81,*p* < 0.01) when compared to the imputed data. There were no other discrepancies in the pattern of results when comparing non-imputed to imputed data.

### Exploratory analyses

Causal mediation analyses were conducted on the primary outcome variables (GAS 1, GAS 2, and PANSS T) to assess whether medication had an indirect influence on the significant changes found from pre- until post-therapy. Analyses were conducted merely for antipsychotics, as only three patients were treated with other psychotropic medications. No significant results were found (see Table 3) for the primary outcome variables.

**TABLE 3:** Non-parametric causal mediation analysis (*n* = 27): indirect effect of antipsychotic medication on primary outcomes.

	Beta	Nonparametric bootstrapping BC 95% CI		*p*
		
		*LL*	*UL*	
**GAS1**				
ACME	−0.28	−1.46	0.17	0.38
ADE	0.45	−0.60	1.33	0.32
Total effect	0.17	−1.75	1.39	0.82
Prop. mediated	−1.67	−5.58	4.04	0.80
**GAS 2**				
ACME	−0.04	−1.20	0.85	0.89
ADE	0.90	−0.28	1.66	0.10
Total effect	0.86	−1.42	2.24	0.31
Prop. mediated	−0.05	−3.44	3.93	0.79
**PANSS T**				
ACME	−0.57	−1.82	0.10	0.11
ADE	−0.05	−1.01	0.64	0.86
Total effect	−0.62	−2.84	0.75	0.50
Prop. mediated	0.92	−4.92	5.80	0.38

GAS, Goal Attainment Scale; ACME, average causal mediation effects; ADE, average direct effects; Prop. mediated, proportion mediated; 1,000 bootstrap samples; CI, confidence interval; LL, lower limit; UL, upper limit.

Among the secondary outcome variables that showed a significant total and specific effect, seven of the variables assessed different aspects of psychotic symptoms (PDI, PSYRATS-D, PSYRATS-H, PCL-F, PCL-C, PANSS-N, and PANSS-P). As the PANSS T score was already included in the mediation analysis, we decided not to conduct further causal mediation analyses for specific psychotic symptoms. Additionally, no causal mediation analyses were conducted for the two other secondary outcome variables with a significant total and specific effect assessing functioning (PANSS-G and RFS), as functioning is also included in the PANSS T score.

Whereas there was a total significant effect for one putative mediator variable (ERSQ), no specific effects were found, which is why no causal mediation analysis was performed.

## Discussion

To our knowledge, this is the first paper examining the feasibility and the potential efficacy of a mindfulness-based inpatient group intervention specifically targeting ER in patients with EP. The main aim of this study was to assess the feasibility and acceptance of the Feel-Good intervention in an inpatient setting. All patients completed the group intervention and 37% of the patients asked to continue to partake in the group. Thus, our findings suggest that a mindfulness-based intervention is feasible and acceptable in inpatient settings specialized on EP. Our feasibility findings are in line with findings from the other eight studies reported in the meta-analysis examining third-wave interventions for individuals with EP, where an average attendance rate of 72.2% (range 56–100%) was reported (53).

Another indicator we used to assess feasibility and acceptance were dropout rates. Aside from the dropouts associated with the COVID-19 pandemic and government restrictions, there were no dropouts in our study between pre- and post-group assessment, suggesting a high acceptability of the group. This is in line with findings reported from the EP meta-analysis, where an average drop-out rate of 18.7% (range 0–37.5%) was reported (53). The dropout rates between post-group-assessment and FU-assessment in our study could be attributed to numerous factors: (1) patients reported that they focused on re-integrating into academics/job, (2) reduction of stress (not undertaking too many activities after hospital discharge), and (3) participation of assessment measures was only possible during working hours/when classes took place. Thus, we assume that dropouts were not linked to a dislike toward the Feel-Good intervention.

The secondary aim was to gather initial findings regarding the efficacy of the Feel-Good intervention. We found that there was a statistical change in terms of patients’ individual goal attainment (reaching one’s emotional goals). Thus, patients in this study reported an improvement in obtaining their emotional goals at post-group and FU time points. To our knowledge, no other studies have utilized the GAS to assess change in reaching one’s emotional goals in patients with EP nor with patients with psychotic disorders in general. Therefore, we are not able to compare our results with other studies with regards to goal attainment. Yet, results suggest that mindfulness-based interventions may be helpful in terms of reaching one’s emotional goals.

We also found a significant statistical change in the second primary outcome overall psychotic symptoms. Patients reported a reduction in psychotic symptoms. Furthermore, patients reported a significant statistical change and reduction in specific psychotic symptoms, specifically in delusions, hallucinations and negative symptoms. Only two of the eight studies examined in the EP meta-analysis reported pre–post within-group changes of psychotic symptoms. One study did not find any significant changes in the PANSS scales between pre- and post-assessment (89). The other study found significant changes in the PANSS total score (*r* = 0.66), albeit no significant changes on the PANSS positive nor PANSS negative subscales (90). Whereas our study found an overall large effect on psychotic symptom reduction, effects in specific psychotic symptoms ranged from small to large effects. Of interest, is that Khoury et al. (13) found higher effects on negative symptoms compared to positive ones, which is not what our findings reveal. In fact, when examining our non-imputed data there was no significant change found in negative symptoms after the intervention. Differences in our findings may be attributed to patients in our study being less affected by negative symptoms and being more affected by positive symptoms.

When examining other clinical symptoms, no significant change was found for depressive symptoms. Findings from three studies included in the EP meta-analysis found significant within-group reduction in depressive symptoms (90–92). The fact that we did not find any significant changes might be due to relatively low depression scores (CDSS) pre-group intervention; thus, there was little room for significant improvement. We did find significant changes in role functioning. None of the studies included in the EP meta-analysis reported within-group pre–post-assessments of role functioning. However, one study did report a significant improvement in the quality of life (*r* = −0.59), which can be associated indirectly to functioning (90). General psychopathology also showed significant changes in our study, which corroborates the significant findings of Tong et al.’s (90) study (*r* = 0.70)

There was an absence of significant effects on most putative mediators (ER). The only significant change after the Feel-Good intervention was in terms of how individuals assessed their own emotional regulation skills (with a change directed toward improvement) within the past week. None of the studies in the EP meta-analysis assessed ER, thus, we have no comparison within the field of EP. Our findings do support another study that utilized ERSQ in their research with an emotion-oriented individual CBT intervention targeting ER in individuals with psychosis in an inpatient setting (62). Mehl et al. (62) also found significant improvements with a small effect size (*d* = −0.15).

An exploratory analysis was conducted to assess whether significant changes found in psychotic symptoms and emotional goal attainment were attributed to changes in medication. Our mediation analyses suggests that this was not the case. Thus, our results suggest that effects of the mindfulness-based intervention “Feel-Good” may not be attributed to antipsychotic medication. Yet, as this analysis is conducted based on a single experimental condition no causal conclusions can be drawn.

Interestingly, we found a general effect in one out of six questionnaires assessing ER, but there was only a total effect over all three assessment time points and not a specific effect, thus, we did not perform a mediation analysis. Concluding, our results partly suggest that ER might indirectly improve psychotic symptoms, but the results are somewhat inconclusive.

Taken together, the effects produced by the Feel-Good intervention ranged from having large effects on improving emotional goals, psychotic symptoms, general psychopathology, and everyday functioning, whereas having small to no effects on potential mediators. The initial findings of this pre–post study suggest that mindfulness-based interventions may be helpful in reducing delusions and achieving better goal attainment in terms of emotion. Yet, a large-scale RCT including a control condition that receives routine care needs to be conducted for more conclusive findings. Furthermore, we found a small inconclusive effect for ER, but it remains unclear what role the putative mediator plays in changes seen in the psychotic symptoms and emotional goal attainments. Thus, again, future RCTs are required to understand the relationship better and draw more conclusive findings.

Numerous reasons can explain the absence of clear intervention effects of mediators. Each group session had its own focus and it was not possible to practice many of the skills, which may have benefited the patients more. For example, in module 3 patients were told about and practiced mindfulness technique. However, in module 4, a mindfulness exercise was practiced at the start of the session and then new strategies were introduced on how to reduce vulnerability toward negative emotions in general. Thus, there was a lot of information for patients who do suffer from acute psychotic symptoms, and not a lot of time to practice individual strategies discussed in each module. A more promising approach may be to extend the group intervention to more sessions that include more room for practical exercises.

Additionally, individuals reported different emotions as goal attainment targets. Whereas there was a great overlap in some emotions (i.e., anger, sadness, and loneliness), the Feel-Good intervention was only able to delve into two specific emotions to discuss how specific techniques can be applied to real-life situations. For some individuals, these emotions were not a priority or regarded as problematic, thus, they were less likely to benefit from these sessions and lacked more “hands-on” examples for the emotions they deemed as problematic. Thus, a more focused intervention concentrating including more sessions on specific emotions and ER strategies may be deemed as more helpful and beneficial. Furthermore, it should be contemplated whether the group intervention should be offered over a longer time period and thus, accompany patients after they are discharged, as problems with negative emotions may arise more frequently in real-life settings.

Another reason as to why little to no effects were found for potential mediators may be because a large proportion of our participants (40%) were diagnosed with a substance-induced psychotic disorder. Research has found that for many individuals with psychotic disorders, substances are used to cope with difficult emotions (93). Therefore, individuals may have overestimated their abilities to regulate their emotions, as they may be more likely to suppress/avoid negative emotions through substances and thus, experience them for shorter periods of time than people who may not suppress/avoid them.

### Limitations and strengths of the study

The current study has the following limitations. First, our pilot study sample size was small and thus inferences should be interpreted cautiously. Furthermore, we adjusted our statistical analyses and did not perform a power calculation. Thus, both the negative and positive findings should be interpreted with caution. Despite this limitation, the current results did find a large effect size in terms of improvements in psychotic symptoms, general psychopathology, as well as attaining ones’ emotional goals in a pre–post-design that allows cautious conclusions on the interventions efficacy that needs to be confirmed in a RCT design.

Secondly, the absence of a control group does not allow for any causal interpretations to be made on the observed changes. Thus, no attributions can be made solely to the Feel-Good intervention per se. However, as our results resemble the findings reported in other studies who targeted potential putative mediators of psychotic symptoms, it is unlikely that similar results stem from treatments implemented in routine clinical care.

A further limitation of the study is the heterogeneity of our sample, as it cannot be ruled out entirely that different diagnoses can have different prognoses. However, studies have found that diagnostic transitions from substance-induced psychotic disorders to more severe illnesses (i.e., schizophrenia) occur frequently in individuals aged 16–25 and that there were no significant differences in the prognoses of individuals with substance-induced or non-substance-induced EP (94, 95).

Another limitation is that the primary outcome (emotional goal attainment) is a very patient-and outcome-centered approach, thus, there are potential restrictions in terms of its validity and reliability. Yet, a systematic review identified the GAS as the most common measure for goal attainment in therapy that has the strongest evidence for its clinical utility (96). Other limitations include the high rate of missing values between post-group and FU, as well as the fact that the raters and group therapists were not blinded.

Lastly, a further limitation was that attendance rates of individual and group therapy sessions as part of treatment as usual (TAU) were not measured. Therefore, improvements measured in overall symptomology found in this study cannot be solely attributed to the Feel-Good Therapy. It is therefore essential to control attendance rates of individual and group therapy sessions for TAU in larger RCT studies.

## Conclusion and implications

The results suggest that a mindfulness-based intervention is feasible in a group format in inpatient settings. Furthermore, the attendance rates and reasons for drop-out between post intervention and follow-up suggest that the group was highly acceptable for the patients with EP. The results also show a positive change on both primary outcomes (goal attainment and psychotic symptoms) for patients with EP that we can cautiously attribute to some degree to the mindfulness-based “Feel-Good” intervention. Our results did not reveal a significant change in putative mediators (ER and negative self-schemata) after the mindfulness-based intervention, besides in one scale pertaining to self-perception of ER skills.

Future research is essential to assess whether improvements in the treatment manual and training may lead to stronger changes in putative mediators and thus stronger effects on psychotic symptoms. To achieve a stronger effect on the putative mediators, a more targeted approach may be a more plausible course of action (using less interventions). Also, more time should be allocated for patients to practice the newly acquired skills outside of the group, so that they can gain more experiences implementing the skills in their everyday lives, which may lead to greater changes in the putative mediators. These changes should be adapted and implemented in the Feel-Good group intervention, so that a large-scale RCT can be conducted to see whether (1) changes found in this study remain significant when compared to a control group receiving routine care, and (2) whether there were any significant changes in putative mediators.

Continuity of care between inpatient and outpatient settings are essential, especially for patients with psychosis, as it may prevent detrimental trajectories of the illness and be helpful in regaining quality of life (97). Thus, implementing the Feel-Good Group Therapy into outpatient settings may be a sequential step when treating patients with psychosis. As outpatient settings have less of a time limit, it may be beneficial to extend the Feel-Good intervention beyond eight sessions, which also allows for more in-depth exploration of certain topics or themes (i.e., individual emotions) that appeal to the individuals attending the group therapy. Furthermore, this allows for more repetition of the concepts discussed in the group and more time and practice to implement these concepts in their everyday life. The Feel-Good intervention can either be combined with individual therapy or applied after individual therapy is completed. This is especially the case for patients with psychosis who feel socially isolated and battle with loneliness. Lastly, it may be important to offer booster sessions to ensure long-term stability. This can either be done by having the same group meet after a certain amount of time, by offering an individual session, or by having individuals from a completed intervention join a currently running Feel-Good intervention.

## Data availability statement

The original contributions presented in this study are included in the article/Supplementary material, further inquiries can be directed to the corresponding authors.

## Ethics statement

The studies involving human participants were reviewed and approved by the Psychologische Hochschule Berlin. Written informed consent to participate in this study was provided by the participants’ legal guardian/next of kin.

## Author contributions

All authors listed have made a substantial, direct, and intellectual contribution to the work, and approved it for publication.
