# Supplementary material for: Feasibility and efficacy of an acceptance and mindfulness-based group intervention for young people with early psychosis (Feel-Good group)
Source: Front Psychiatry. 2022 Sep 16;13:943488. doi: 10.3389/fpsyt.2022.943488 (PMC9522968; doi:10.3389/fpsyt.2022.943488)
Supplement: Supplementary file 1 [file Table_1.DOCX]

Supplementary Material: *Feasibility and Efficacy of a Mindfulness-based Intervention for Early Psychosis*

**Table S1:** Intervention Overview

| **Session Number** | **Topic/Intervention** |
| --- | --- |
| 1 | **Introduction of the Feel-Good group:** Introduction to therapists and participants. An overview of the Feel-Good group modules is provided. The group establishes group rules for later sessions (i.e., respect for others). Individual emotion therapy goals of each patient are discussed. Expectations of the group intervention are discussed. |
| 2 | **Module 1:** Information on emotions, their biological functioning evolutionary aspects are discussed. Connections between emotions and mental health are explored. |
| 3 | **Module 2:** Patients are asked to share experiences of negative emotions and how they dealt with them. The group then discusses general strategies/techniques/skills patients used to cope with negative emotions to improve emotional awareness and to explore, how patients’ thoughts influence negative emotions. The concept of mindfulness is introduced as an emotion regulation strategy. |
| 4 | **Module 3:** Introduction of the basic concepts of mindfulness (i.e. focused attention, being in the present, non-judgmental acceptance). A guided imaginary mindfulness exercise is practiced, which is then followed by reflections on the exercise. |
| 5 | **Module 4:** A mindfulness exercise is practiced, later the group reflects on how they feel after the exercise. Difficulties are identified and perceived benefits of the exercise are discussed. Then, patients learn general skills on how to reduce their vulnerability towards negative emotions. Patients receive additional information on the necessity of drug abstinence, daily routines and positive activities to reduce their general vulnerability towards negative emotions. |
| 6 | **Module 5:** A mindfulness exercise is practiced. Then, the session focuses on conveying strategies to cope with anger. Thoughts that arise in relation to the emotion anger are discussed. Patients are then guided into reappraising automatic thoughts previous to situations in which they perceive anger and during the situations. They discuss their automatic thoughts in the group, reflect on them and develop more functional self-instructions that can be applied in future situations that are related to the emotion anger. Patients also discuss on how to change their behavior, i.e.. by applying mindfulness practice to real-life situations, which resulted in anger to see how applying mindfulness strategies can reduce anger. |
| 7 | **Module 6:** A mindfulness exercise is practiced. This session focuses on conveying strategies to cope with the emotions shame and guilt. Automatic thoughts that arise when patients are feeling guilty or ashamed are discussed, reflected and more functional automatic thoughts or self-instructions are produced. Patients are then guided into applying mindfulness practice to real-life situations, which resulted in guilt or shame to see how applying mindfulness strategies can influence shame and guilt. |
| 8 | **Module 7:** A mindfulness exercise is practiced. All the sessions are then reviewed; therapists go through each practice component. Discussion of future plans for continuing cognitive reappraisal and mindfulness as a strategy to cope with negative emotions is discussed. Patients devise their own ‘emergency plans’ that contain steps taken once they notice a worsening in their emotional states or psychotic symptoms. Closing Celebration |

**Table S2:** Change in primary and main secondary outcome variables and putative mediators between pre-, post- and Follow-Up assessment (sample as treated; *n*=21)

| Measure | T1 Scores  *M (SD) Mdn* | T2 Scores  *M (SD) Mdn* | T3 Scores  *M (SD) Mdn* | Timepoint Differences | | | | Effect Sizes | | Pairwise Comparisons*  Pre-Post Post-FU- Pre-FU Assessment Assessment Assessment | | | | | | |  |
| --- | --- | --- | --- | --- | --- | --- | --- | --- | --- | --- | --- | --- | --- | --- | --- | --- | --- |
|  |  |  |  | $\chi^{2}$ | *df* | *p* | W | | 95% CI | | $Z$ | *p* | $Z$ | *p* | $Z$ | *p* | |
| *Primary Outcome Variables* | | | | | | | | | | | | | | | | |  |
| GAS 1 | -1.29 (0.78) -1.00 | -1.48 (0.75) -2.00 | 0.81 (1.17) 1.00 | 33.3 | 2 | <.001 | .79 | | 0.68-0.90 | | -4.26 | <.001 | -2.55 | .011 | -3.97 | <.001 | |
| GAS 2 | -1.48 (0.75) -2.00 | .24 (1.58) 0.00 | .76 (1.38) 1.00 | 30.00 | 2 | <.001 | .71 | | 0.54-0.89 | | -4.10 | <.001 | -2.21 | .027 | -3.88 | <.001 | |
| PANSS T | 68.43 (18.12) 66.00 | 59.00 (16.19) 56.00 | 50.76 (14.73) 51.00 | 31.14 | 2 | <.001 | .74 | | 0.59-0.91 | | -3.26 | .001 | -3.39 | <.001 | -4.02 | <.001 | |
| *Secondary Outcome Variables* | | | | | | | | | | | | | | | | |  |
| PANSS-P | 17.14(6.00) 18.00 | 13.05 (4.84) 12.00 | 10.62 (4.20) 10.00 | 25.31 | 2 | <.001 | .60 | | 0.44-0.80 | | -3.79 | <.001 | -3.18 | .001 | -3.73 | <.001 | |
| PANSS-N | 16.14 (8.57) 12.00 | 15.05 (5.12) 14.00 | 13.43 (5.46) 13.00 | 5.84 | 2 | .054 | .14 | | 0.03-0.40 | | -1.23 | .217 | -1.88 | .060 | -2.47 | .010 | |
| PANSS-G | 35.14 (6.36) 34.00 | 30.90 (7.58) 30.00 | 26.81 (6.9) 25.00 | 22.30 | 2 | <.001 | .53 | | 0.32-0.78 | | -2.55 | .011 | -2.86 | .004 | -3.87 | <.001 | |
| PCL-F (P) | 25.57 (17.00) 23.00 | 14.38 (15.68) 10.00 | 11.10 (14.70) 6.00 | 17.84 | 2 | <.001 | .43 | | 0.19-0.74 | | -3.19 | .001 | -2.10 | .036 | -3.47 | <.001 | |
| PCL-C (P) | 27.86 (18.52) 27.00 | 16.00 (15.56) 12.00 | 11.86 (14.06) 4.00 | 23.73 | 2 | <.001 | .57 | | 0.34-0.80 | | -3.37 | <.001 | -2.00 | .046 | -3.62 | <.001 | |
| PDI T | 67.38 (52.42) 52.00 | 34.00 (43.55) 20.00 | 21.71 (37.43) 7.00 | 35.19 | 2 | <.001 | .84 | | 0.70-0.97 | | -3.87 | <.001 | -3.09 | .002 | -4.02 | <.001 | |
| PSYRATS-D | 14.62 (6.77) 15.00 | 7.62 (7.26) 8.00 | 5.86 (6.38) 4.00 | 25.87 | 2 | <.001 | .62 | | 0.32-0.91 | | -4.04 | <.001 | -2.52 | .012 | -3.69 | <.001 | |
| PSYRATS-H | 7.14 (12.61) 0.00 | 12.05 (15.08) 0.00 | 5.67 (10.97) 0.00 | 12.41 | 2 | .002 | .30 | | 0.14-0.53 | | -2.39 | .017 | -2.02 | .043 | -2.67 | .008 | |
| RFS | 39.90 (9.27) 42.00 | 38.86 (9.14) 38.00 | 43.24 (9.03) 45.00 | 7.90 | 2 | .019 | .19 | | 0.05-0.45 | | -.45 | .657 | -2.78 | .005 | -2.21 | .027 | |
| CDSS | 4.62 (3.28) 5.00 | 5.24 (3.66) 4.00 | 4.10 (4.21) 2.00 | 1.92 | 2 | .382 |  | |  | |  |  |  |  |  |  | |
| PCL-D (P) | 33.05(20.74) 42.00 | 19.81 (19.88) 15.00 | 17.71 (19.64) 6.00 | 4.53 | 2 | .104 |  | |  | |  |  |  |  |  |  | |
| *Putative Mediators* | | | | | | | | | | | | | | | | |  |
| ERSQ (P) | 56.81 (24.14) 63.00 | 58.52 (15.95) 61.00 | 62.67 (21.91) 69.00 | 9.81 | 2 | .007 | .23 | | 0.06-0.56 | | -.534 | .594 | -1.86 | .063 | -1.63 | .102 | |
| BASS-N (P) | 23.43(6.08) 25.00 | 22.19 (4.78) 23.00 | 22.05 (4.20) 21.00 | 3.31 | 2 | .191 |  | |  | |  |  |  |  |  |  | |
| BASS-P (P) | 7.90 (3.83) 7.00 | 8.14 (2.18) 9.00 | 8.81 (2.93) 11.00 | 3.65 | 2 | .161 |  | |  | |  |  |  |  |  |  | |
| BASS-C (P) | 6.95 (2.44) 8.00 | 7.57 (1.66) 8.00 | 8.43 (1.81) 8.00 | 3.65 | 2 | .161 |  | |  | |  |  |  |  |  |  | |
| ERI- NE (P) | 36.33 (10.88) 37.00 | 41.00 (11.27) 43.00 | 40.05 (10.88) 42.00 | 4.84 | 2 | .089 |  | |  | |  |  |  |  |  |  | |
| ERI-PO (P) | 22.52 (10.07)26.00 | 21.24 (9.85) 20.00 | 19.14 (7.20) 20.00 | 4.23 | 2 | .121 |  | |  | |  |  |  |  |  |  | |
| ERQ-R (P) | 22.33 (6.57) 23.00 | 24.76 (6.55) 23.00 | 25.90 (4.42) 26.00 | 3.88 | 2 | .144 |  | |  | |  |  |  |  |  |  | |
| ERQ-S (P) | 15.24 (6.62) 16.00 | 13.76 (5.29) 12.00 | 15.43 (4.42) 15.00 | 3.49 | 2 | .175 |  | |  | |  |  |  |  |  |  | |
| RSE (P) | 25.29 (3.07) 26.00 | 26.05 (1.66) 26.00 | 25.67 (2.37) 26.00 | 1.50 | 2 | .472 |  | |  | |  |  |  |  |  |  | |
| SCS (P) | 2.74 (.72) 2.83 | 2.91 (.47) 2.89 | 3.10 (.61) 3.15 | 4.67 | 2 | .097 |  | |  | |  |  |  |  |  |  | |

*Notes:* M = Mean; SD = Standard deviation; df= Degrees of Freedom; W= Kendall’s W; CI= Confidence Interval; GAS = Goal Attainment Scale; PANSS = Positive and Negative Syndrome Scale; PANSS T= PANSS Total score; PCL-F= Paranoia Checklist Frequency; PCL-C = Paranoia Checklist Conviction; P = Self-report Questionnaire; PDI = Peters et al. Delusions Inventory 21 Total score; PANSS P= PANSS positive scale; PANSS N = PANSS negative scale; PANSS G= PANSS general psychopathology scale; PSYRATS-D= Psychotic Rating Symptom Scale Delusions; PSYRATS-H= PSYRATS Hallucinations; RFS = Role functioning scale: RFS mean score= mean score of scales Social Network I, Social Network II, Work and Living; CDSS = Calgary Depression Rating Scale; PCL-D = Paranoia Checklist Distress; ERSQ= Self-Report Measure for the Assessment of Emotion Regulation Skills total score; BASS-N= Beliefs about Stress Scale-Negative; BASS-P= Beliefs about Stress Scale- Positive; BASS-C = Beliefs about Stress Scale- Controllability; ERI-NE = Emotion Regulation Inventory–Negative; ERI-PO= Emotion Regulation Inventory-Positive; ERQ-R=Emotion Regulation Questionnaire-Cognitive Reappraisal; ERQ-S=Emotion Regulation Questionnaire- Expressive Suppression; RSE =Rosenberg Self-Esteem Scale total score; SCS= Self-Compassion scale total score.

*Pairwise Comparisons between the three assessment timepoints were only computed when the Friedman Test revealed significant results.

**
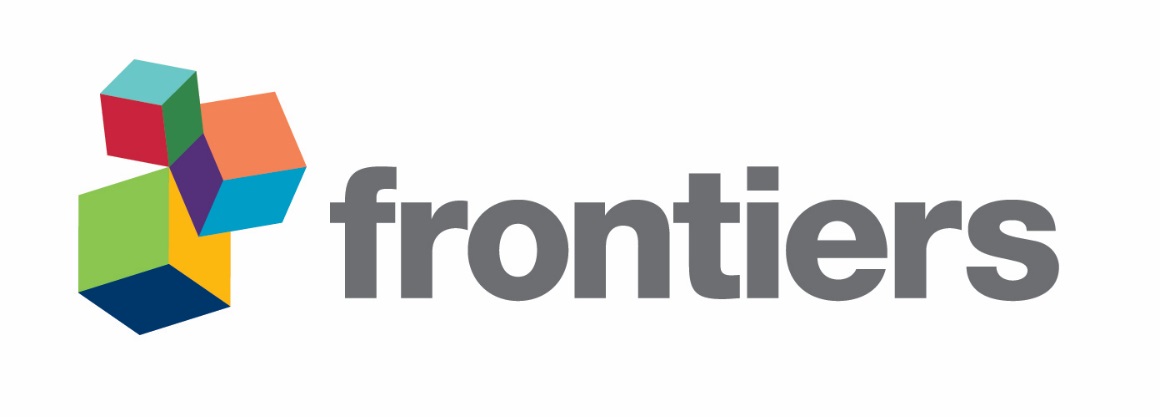
**
